# Searching for Differences in Chemical Composition and Biological Activity of Crude Drone Brood and Royal Jelly Useful for Their Authentication

**DOI:** 10.3390/foods10092233

**Published:** 2021-09-21

**Authors:** Ewelina Sidor, Michał Miłek, Grzegorz Zaguła, Aleksandra Bocian, Małgorzata Dżugan

**Affiliations:** 1 Department of Chemistry and Food Toxicology, Institute of Food Technology and Nutrition, University of Rzeszów, Ćwiklińskiej 1a St., 35-601 Rzeszów, Poland; ewelina.sidor@poczta.onet.pl (E.S.); mmilek@ur.edu.pl (M.M.); 2 Department of Bioenergetics, Food Analysis and Microbiology, Institute of Food Technology and Nutrition, College of Natural Science, Rzeszów University, Ćwiklińskiej 2D St., 35-601 Rzeszów, Poland; g_zagula@ur.edu.pl; 3 Department of Biotechnology and Bioinformatics, Rzeszów University of Technology, Powstańców Warszawy 6 St., 35-959 Rzeszów, Poland; bocian@prz.edu.pl

**Keywords:** drone brood, royal jelly, minerals, sex hormones, antioxidant activity, enzymes, protein profile, HPTLC

## Abstract

Drone brood is a little-known bee product which is frequently considered as a male equivalent of royal jelly and is sometimes used as its adulterant. The aim of the study was to compare the chemical composition and biological activity of both bee products originated from the same apiaries (*n* = 3) limiting the influence of genetic and environmental factors. Moreover, for drone brood study covered testing three stages of larval development (days 7, 11, and 14). The comparison included mineral composition (ICP-OES method), protein content and protein profile (SDS-PAGE), testosterone and estradiol content (ELISA tests). HPTLC method was used to analyze of sugar, amino acids, and polyphenolic profile of drone brood and royal jelly. Moreover, their antioxidant and enzymatic properties were compared. A lot of similarities between drone brood and royal jelly were found in terms of chemical components. However, drone brood was more abundant in iron and manganese, reducing sugars and some amino acids, especially proline, tyrosine, and leucine. It contained more testosterone (especially on the 14th day) and estradiol (on the 7th day). The greatest differences in the enzymatic activities and polyphenolic profile were found. Diastase and α-glucosidase activity were found as specific enzymes of the drone brood. Similarly, ferulic and ellagic acids were characteristic for brood and were not present in royal jelly. The study showed a lot of similar features for both tested bee products, however, some specific markers which can serve to differentiate drone brood and royal jelly were found.

## 1. Introduction

Since the end of the 20th century, beehive products have been more widely used in modern medicine as a curative and preventive remedy. Honey, pollen, royal jelly, and propolis are unique natural products, that contain balanced combinations of the most important biologically active components, which determine a wide range of their medicinal properties. It has been shown that in many biologically active beehive products, royal jelly is the most important one [1]. In turn, drone brood is a valuable bee product little known in the world, however with a long history of application in Romania and Russia [2].

Royal jelly (RJ) is a yellowish-white, creamy, acidic secretion from the mandibular and hypopharyngeal glands of young worker bees (the so-called nurses) of the *Apis mellifera* species [3]. It serves as the most important part of the honeybee larvae diet, including both worker and drone larvae, playing a major role in caste differentiation. This milk is fed to all larvae in the bee colony for the first three days of life, and the queen to the entire larval stage. Scientific researchers have shown that royal jelly is a rich source of protein, carbohydrates, lipids, minerals, and vitamins, as well as female sex hormones. It was shown that royal jelly given to the mother larvae has a richer chemical composition (more sugars, juvenile hormones and acids) than that intended for other larvae [1,3]. Royal jelly lipids consist of fatty acids (90%), sterols, glycerides, and phospholipids. Among sugars, glucose, fructose, sucrose, ribose, and traces of glycogen were found. Proteins, including albumin, globulins, complex proteins, hormones and enzymes, make up the largest share of the composition of RJ. The numerous enzymes are divided into those involved in the synthesis of proteins and carbohydrate metabolism, associated with the oxidation and reduction processes or involved in lipid metabolism and transport. Among them, glycosidases belonging to the hydrolases group, constitute a diverse enzymes complex, related to the biogenesis, transport, and catabolism of glycoconjugates [4,5]. Royal jelly is also rich in macro- and microelements, vitamins of the B group, pantothenic acid, nicotinic acid, and inositol. Organic acids contained in RJ cause acidic conditions (pH 4.1–4.8) [6].

The abundance and variety of bioactive compounds occurring in royal jelly are responsible for its multiple physiologic activities and medical applications. The beneficial effects as a result of the ingestion of RJ through capsules, tablets, or other preparations are based on experimental studies in which the group was treated with a placebo [7]. Several pharmacological properties have been attributed to RJ mainly among which are antioxidant activity, due to hormones, enzymes used in healthy foods, the pharmaceutical industry or cosmetics [8,9].

In turn, drone brood (DB) is a bee product gaining more and more popularity, often referred to as the male equivalent of royal jelly. This assumption is based on the similarity in the chemical composition of both RJ and DB products. However, its origin is completely different. The drone brood is collected from beehives as young male larvae, usually between days 4 and 14 of development [2]. As DB can be easily obtained in large quantities, it is used to counterfeit royal jelly [9,10,11]. 

Drone brood is a viscous yellowish liquid with a pleasant odor, which contains a large number of beneficial substances for humans. The chemical composition of drone brood homogenate is characterized by the presence of proteins, amino acids, nucleic acids, enzymes, and phospholipids. The complex of biologically active substances of drone brood determines the number of pharmacological characteristics of these bee products, in particular, the antioxidant, immunotropic, adaptogenic, and anabolic actions. Drone brood is characterized by the presence of enzymes with a large number of functional groups, as well as hormones [2,12]. Drone brood homogenate is unstable during storage and requires additional stabilization to maintain the biological activity of the active substances for a long time. Different methods are used to stabilize biologically active substances in preparations based on drone brood homogenate. Although drone brood is a little-known product of beekeeping, it has long been used as a cheap, safe, and effective natural remedy against various diseases [13,14]. 

So far, preliminary studies have been carried out on the chemical composition and biological activity of drone brood which prove the high content of nutrients, bioelements, enzymes, and sex hormones. However, there are still no detailed studies of the chemical composition comparing the drone brood to royal jelly which is a well-tested bee product. The aim of the work was to compare the chemical composition of drone brood collected at various stages of development with royal jelly and to find the specific chemical markers to distinguish between the two products.

## 2. Materials and Methods

### 2.1. Chemicals 

The chemicals [2,2-diphenyl-1-picrylhydrazyl; 2′-azino-bis (3-ethylbenzothiazoline- 6-sulfonic acid); 2,4,6-Tris(2-pyridyl)-s-triazine], reagents (Folin–Ciocalteu reagent), standards: ferulic acid, ellagic acid, proline, tyrosine, glycine, lysine, histidine, leucine, aspartic acid, valine were obtained from Sigma Aldrich (St., MO, USA), Elisa kits for testosterone and estradiol from Abbexa (Cambridge, UK) and buffer components (chloroform, ethyl acetate, formic acid, ethanol, 1-butanol, 2-propanol, boric acid) were purchased from Avantor Performance Materials Poland SA (APM, Gliwice, Poland).

### 2.2. Material Collection

**Figure 1:**
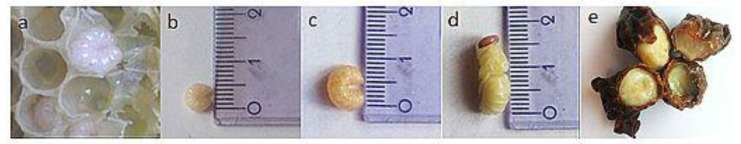
The size of drone larvae at various stages of development: (**a**) 7-day male larva in the comb cell, (**b**) 7-day-old larva, (**c**) 11-day-old larva, (**d**) 17-day old pupa, (**e**) royal jelly.

Samples of drone brood (7-, 11-, 14-day-old; *n* = 9) and royal jelly (*n* = 3) were collected from the three apiaries (localized at least 50 km apart) in the south-eastern part of Poland (50.31° N, 21.26° E; 50.03° N, 22.93° E; 50.30° N, 22.25° E) in the June 2020 season. The drone brood (50 g from each sample) of the *Apis mellifera carnica* breed families were selected by hand from the drone frame, immediately sealed in sterile containers and transferred to the laboratory. The 7-, 11- and 14-day-old larvae were collected (Figure 1).

Each sample was homogenized using a tissue homogenizer (TH 02, Omni International, Kennesaw, GA, USA) with 7 mm Omni Tips ™ plastic tips. The material was then frozen at −18 °C. The collection of royal jelly consisted in extracting the queen cells from the whole combs, which was then cut, and royal jelly was collected with a metal spatula into Eppendorf tubes. The material was immediately frozen at −18 °C.

### 2.3. Preparation of Drone Brood and Royal Jelly Extracts 

Half a gram of frozen drone brood or royal jelly sample was extracted with 5 mL of distilled water. The samples were homogenized with a tissue homogenizer (TH 02, Omni International, USA) for two minutes at medium speed (15,000 rpm). The extracts were then centrifuged for 20 min at 10,000 rpm at 4 °C using a refrigerated centrifuge (MPW-351R, Med. Instruments, Warszawa, Poland). The supernatants were collected and then filtered through a 0.45 µm nylon syringe filter and stored in a refrigerator at 4 °C until further analysis but not longer than 3 days. Before antioxidant activity tests, the extracts were diluted 10-fold. 

### 2.4. Determination of the Physicochemical Properties of Drone Brood and Royal Jelly

#### 2.4.1. Water Content

One gram of the analyzed bee product was weighed on an aluminum plate. Water content was determined in a moisture analyzer (MA 50/1.R, RADWAG, Radom, Poland). A gentle drying profile of 60 °C with an accuracy of 1 mg/25 s was set. The measurement was performed in triplicate.

#### 2.4.2. Refractive Index

The determination of the refractive index was done by the refractometric method, using an electronic refractometer HI96800 (Hanna Instruments, Woonsocket, RI, USA). The value of the refractive index was determined at room temperature with accuracy to the third decimal place. The determinations were made in triplicate.

#### 2.4.3. Active and Free Acidity

To determine active acidity, a pH measurement of 20% solutions of drone brood and royal jelly in distilled water was performed using a CP-401 pH meter (Elmetron, Zabrze, Poland). To determine the free acidity, 50 mL of 20% appropriate extract was titrated by 0.1 M NaOH to reach a pH of 8.3 measured by pH meter. The results were expressed in mval/g (mL 0.1N NaOH/g) of wet weight (WW).

#### 2.4.4. Conductivity

To determine the specific electrical conductivity, 20% solutions of drone brood and royal jelly in distilled water were used. The conductivity of each sample was determined by immersing the electrode in the test solution. Each sample was measured three times. The conductivity of each product solution was measured using a conductometer CP-401 (Elmetron, Zabrze, Poland) and the results (in mS/cm) were presented.

#### 2.4.5. Protein Analyses

##### Total Protein

Total protein was determined based on nitrogen content using carbon/hydrogen/nitrogen analyzer TruSpec (LECO, Saint Joseph, MI, USA), which is based on the Dumas dry combustion technique. The homogenized material was directly suspended to CHN analyzer according to ISO 16948:2015-07. Obtained nitrogen percentage (N) was calculated into protein content (%) by an adequate multiplier 6.25 [15,16].

##### Soluble Protein Fraction

Soluble protein fraction was determined by Bradford method in DB and RJ extracts prepared according to Latimer [16]. Appropriately diluted samples (5 µL) were combined with 250 µL Bradford reagent (G-250) in a 96-well microplate. Samples were incubated for 5 min at room temperature and the absorbance was read at 595 nm using a microplate reader (EPOCH 2, BioTek, Winooski, VT, USA). The results were calculated on the basis of a calibration curve 0–100 µg/per sample (y = 0.0551x, R^2^ = 0.9991). Bovine albumin was used as a standard protein.

##### Protein Profiling by SDS-PAGE

For protein precipitation with acetone, 1 g of drone brood or royal jelly was diluted with deionized water in 1:1 proportion (*w/w*) and 5 volumes of pure acetone were added. After 12 h of precipitation at −4 °C, tubes were centrifuged for 30 min at 10,000× *g* and pellets were dried in the open air. For salting out, samples were five times diluted with deionized water (*w/w*) and precipitated on a magnetic stirrer with the use of solid ammonium sulfate up to the final concentration of 4 M. The samples were then centrifuged for 30 min at 10,000× *g*. Dialysis was performed on samples diluted with deionized water in 1:1 proportion (*w/w*) in D-Tube™ Dialyzer Maxi (MWCO 3.5 kDa, Merck Millipore, Burlington, MA, USA) for 24 h against deionized water, changed every 8 h. All samples after isolation were finally dissolved in 500 µL of standard Laemmli buffer and protein concentration was measured using 2D Quant Kit (80-6483-56, GE Healthcare, Little Chalfont, UK) in two technical repeats with the BSA as a standard in accordance with the manufacturer’s instructions. Samples containing 25 µg of protein were incubated for 5 min at 95 °C and after cooling were applied to 17.5% denaturing gels (with 5% stacking gels). Electrophoresis was carried out initially at 150 V (15 min) and then at 250 V for 2.5 h with cooling on an Omni PAGE WAVE Maxi apparatus (Cleaver Scientific Ltd., Rugby, Warwickshire, UK) according to the standard method of Laemmli. After electrophoresis, all gels were stained with colloidal Coomassie Brilliant Blue G-250 overnight and then washed for 24 h with deionized water in order to remove the remains of the dye. Gels were scanned with Image Scanner III (GE Healthcare) and processed by LabScan 6.0 (GE Healthcare). Analysis of the gels was performed in ImageJ (1.52a) software by generating graphs representing each lane on the gel [17,18].

#### 2.4.6. Mineral Composition of Bioelements Using the ICP-OES Method

Assessment of selected minerals (Na, K, Ca, Mg, P, S, Fe, Mn, Zn, Cr, Cu, Sr, As) and the toxic metals (Al, Cd, Pb) were determined by optical emission spectrometry with inductively-induced plasma (ICP-OES) using a Thermo iCAP 6500 spectrophotometer (Thermo Fisher Scientific Inc., Waltham, MA, USA). The detection limit for each element was determined at a level that was not lower than 1 µg/L. A curve fit factors (R^2^) for all the elements studied were above 0.99. All the analyses were undertaken in three independent replications for each sample. The targeted repeatability expressed as the relative standard deviation (RSD) and targeted recovery were 92–106%, respectively. The method was validated using certified reference material (INCT-TL-1 tea leaves and NIES CRM No. 7 Tea Leaves). The response from the equipment was periodically checked with known standards. In order to identify the relevant measurement lines and avoid possible interferences, the method of adding an internal standard was applied. Yttrium and ytterbium ions were used as internal standards. 

### 2.5. Hormonal Activity Determination

The activity of testosterone and estradiol in tested bee products was demonstrated with the use of immunoenzymatic ELISA Test Kits (abx574169 for estradiol and abx574314 for testosterone), strictly according to the manufacturer’s manual (Abbexa, Cambridge, UK). In total, 50 µL of appropriately diluted sample was added into the test sample wells. Immediately after, 50 µL reagent A was added. After an hour of incubation at 37 °C and a three-time wash with buffer, 100 µL of reagent B was added. The plate was incubated for another 45 min at 37 °C. The washing process was repeated (5 times), then 90 µL of TMB substrate was added to each well and incubated at the same temperature for 20 min, protected from light. A 50 µL stop solution was added and the absorbance was immediately measured at 450 nm [19,20]. 

### 2.6. Antioxidants Assay

#### 2.6.1. DPPH Test

The inhibition of DPPH (2,2-diphenyl-1-picrylhydrazyl) radicals was measured according to the method previously used in our laboratory for honey analysis with slight modifications. In total, 20 µL of the appropriately diluted extract was mixed with 180 µL of DPPH radical methanolic solution (0.1 mM) and kept in the dark for 30 min. After incubation, the absorbance was measured at 517 nm in a microplate reader EPOCH 2. The reduction of DPPH radicals was calculated using the following equation:

AA% = [(Ao − As)/Ao] × 100,

where Ao is the absorbance of the control and As is the absorbance of the tested samples. As positive control Trolox methanolic solution was applied (exhibited 56.5% scavenging activity in 10 mg/mL concentration). 

#### 2.6.2. FRAP Test

The FRAP (Ferric Reducing Antioxidant Power) test was performed according to Dżugan et al. [21]. The FRAP reagent contained 2.5 mL of a 10 mM solution of 2,4,6-Tris(2-pyridyl)-s-triazine (TPTZ) in 40 mM HCl, 2.5 mL of 20 mM FeCl_3_ and 25 mL of 0.3 M acetate buffer (pH 3.6). To 20 µL of diluted extract, 180 µL of FRAP reagent was added and after incubation at 37 °C for 10 min the absorbance of the reaction mixture was measured spectrophotometrically at 593 nm (EPOCH 2) against a blank. Results were expressed as mmol Trolox equivalents (TE) per 100 g of appropriate sample (mmol/100 g) from the calibration curve prepared for Trolox in the range 5–60 nmol/mL (y = 0.152x, R^2^ = 0.9989).

#### 2.6.3. Total Phenolic Content (TPC) Determination

The total content of phenolic compounds was determined using the Folin–Ciocalteu reagent according to Dżugan et al. [21] adapted to use microplates analyses. To 20 µL of diluted appropriate extract 100 µL of 10% Folin–Ciocalteu reagent was added followed by 80 µL of 7.5% (*w/v*) sodium carbonate solution. The samples were kept in the dark for 60 min and then the absorbance was measured against the blank at 760 nm using microplate reader EPOCH 2. The total content of phenolic compounds was expressed in mg of gallic acid equivalents (GAE) per 100 g of appropriate sample (mg GAE/100 g). The results were calculated based on a calibration curve prepared for gallic acid in the range 0–125 µg/mL (y = 0.336x, R^2^ = 0.9914).

#### 2.6.4. Total Flavonoid Content (TFC) Determination 

The total content of flavonoids in the appropriate extracts was assessed using the method of Biju [22]. In total, 100 µL of the undiluted extract was mixed with 100 µL 2% AlCl_3_ (in methanol). The reaction mixture was incubated for 10 min at room temperature until the completion of the reaction. The absorbance of the solution was then measured at 415 nm with a microplate reader EPOCH 2 against methanol blank. The total content of flavonoids in the extracts of drone brood was expressed in mg of quercetin equivalent (QE) per 100 g of brood sample (mg QE/100 g). The results were calculated on the basis of a calibration curve prepared for quercetin in the range 0–125 µg/mL (y = 0.0655x, R^2^ = 0.9999).

### 2.7. HPTLC Analysis

Comparative analysis of polyphenolic, sugar and amino acid profiles for water extracts of the drone brood and royal jelly samples were performed on HPTLC Silica Gel 60 F_254_ plates (20 × 10 cm) purchased from Merck (Darmstadt, Germany). Chosen extracts of drone brood and royal jelly showing the best results in previous analyses were applied to the plate (40 µL for polyphenolic, 5 µL for sugars and amino acids) as 10 mm bands from the lower edge of the plate at the rate of 100 nL/s using a semi-automated HPTLC application device (Linomat 5, CAMAG, Muttenz, Switzerland) (Table 1). 

The chromatographic separation was performed in a chromatographic tank saturated for 20 min with the mobile and developed to a distance of 70 mm. The obtained results were documented using an HPTLC imaging device (TLC Visualizer, CAMAG) under white light, UV 254, and 366 nm. In addition, each plate was derivatized using an automated derivatizer of TLC plates (CAMAG Derivatizer). After derivatization, the plates were imaged under white light and 366 nm. The obtained chromatographic images were analyzed using the HPTLC software (Vision CATS, CAMAG).

### 2.8. Enzymatic Activity Assay

The activities of the following acid glycosidases in water extracts of the drone brood and royal jelly samples were determined: N-acetyl-D-hexosaminidase (HEX), α-D-galactosidase (α-GAL), β-D-galactosidase (β-GAL), α-D-mannosidase (α-MAN), β-D-mannosidase (β-MAN), α-D-glucosidase (α-GLU), β-D-glucosidase (β-GLU). Moreover, acid and alkaline phosphatases were tested. Enzymatic assays were performed according to the method described by Barrett and Heath [23] based on absorbance measurements at 400 nm of enzymatically released p-nitrophenol (p-NP) from suitable p-NP-glycosides (Sigma Aldrich, St., MO, USA). Briefly, mixtures containing 50 µL of diluted enzyme sample and 50 µL of 2 mM appropriate p-NP substrate in 0.2 M citrate buffer at optimum pH (4.0 for β-GAL, α-GAL, and β-GLU; 5.0 for HEX, α-MAN, β-MAN, α-GLU, and acid phosphatase) were incubated at 37 °C. For alkaline phosphatase determination 0.1 M glycine/NaOH buffer pH 10.5 was used. The reaction was terminated by the addition of 1M Na_2_CO_3_/NaHCO_3_ (250 µL) or in the case of acid and alkaline phosphatase 0.25 M and 0.1 M NaOH, respectively. Absorbance was measured using microplate spectrophotometer EPOCH 2 at 400 nm. Results were expressed as enzymatic units U (μmol/g/min). One unit (U) was defined as the enzyme activity hydrolyzing 1 µmol of substrate per min at 37 °C at the optimal pH conditions [23].

The optimum pH for the tested glycosidases was determined by incubating diluted samples of royal jelly or drone brood with the substrate and 0.1M citrate buffer of pH ranging from 2.5 to 7.0 at 37 °C for 10–60 min. After this time, the reaction was stopped by the addition of 1 M carbonate buffer. Absorbance was measured using microplate spectrophotometer EPOCH 2 at a wavelength of 400 nm [23].

The thermostability of selected enzymes from the brood and royal jelly was determined by mixing 1 mL of the respective sample with 1 mL of buffer (0.1 M citrate buffer, pH 4.0 and 6.0). The settings were incubated for 120 min in a bath (60 °C), with 250 µL of mixture taken before insertion into the bath. The same aliquots were taken after 5, 15, 30, 60, and 120 min of incubation consecutively and were cooled in ice water at once. Then, the activity in all collected fractions was determined in standard conditions [23].

α-amylase was determined by a spectrophotometric method with the Phadebas Diastase test (Magle AB, Lund, Sweden) according to the manufacturer′s instructions. A total of 5 mL of a 1% appropriate bee product solution in 0.1 M acetate buffer pH 5.0 was incubated for 5 min at 40 °C in a water bath. A Phadebas Diastase test tablet was then added to each sample and after thorough mixing, incubated at 40 °C for 30 min. Then, 1 mL of 0.5 M NaOH was added, mixed, and filtered into tubes and the absorbance of the filtrate was measured at wavelength 620 nm against a blank (acetate buffer) using a Biomate 3 spectrophotometer (Thermo Scientific, Waltham, MA, USA). One unit (U) was defined as the enzyme activity causing the increase of the absorbance of 0.1 in experimental conditions. 

### 2.9. Statistical Analysis

All calculations were made in triplicate unless otherwise indicated. For the obtained data, mean values were calculated. Significant differences were calculated by one-way analysis of variance followed by Tukey’s test of significant difference (*p* < 0.05). All calculations were made using the Statistica 13.3 software (StatSoft, Tulsa, OK, USA).

## 3. Results

### 3.1. Physicochemical Properties of Drone Brood and Royal Jelly

In the first step of the study, the physicochemical properties of crude drone brood and royal jelly collected from three apiaries were determined. The comparison covered DB at three different stages of larval development (days 7, 11, and 14). The results are presented in Table 2. 

In this report, the analyzed bee products showed a slightly different water content, the mean value for drone brood was 3.4% higher than in the royal jelly (*p* < 0.05). Moreover, the 14-day-old drone brood showed the highest content of water. Significant differences were found in the activity of hydrogen ions (pH) in the tested products. Drone brood showed significantly higher pH compared to royal jelly (*p* < 0.05). The highest pH was found for DB in 11 day of development, however, the development stage did not influence the pH of the DB samples significantly. A similar relationship was observed for the conductivity of the extracts. Drone brood was characterized by a significantly higher acidity by over 50% compared to royal jelly, and the differences regarding the development stage were not statistically significant. Checking the refractive index, the differences were found between DB and RJ samples only, but the differences were not statistically significant (*p* > 0.05).

The research showed that both drone brood and royal jelly are rich sources of bioelements. However, the royal jelly contained slightly higher values for all tested minerals, excluding iron and manganese. Among the identified macroelements, the highest content in DB was found for phosphorus and potassium. In the analyzed bee products, comparable contents were found for sodium, calcium, and magnesium, however, the differences were statistically significant (*p* < 0.05). The lowest value of macronutrient in drone brood was found for sodium. Among microelements, the highest level was found in the case of zinc. Slightly lower concentrations of manganese and iron were identified, while no iron was found in royal jelly. In addition, trace amounts of contaminants such as lead and cadmium were identified in the drone brood, and a higher content was found in the case of aluminum similar in both tested products.

### 3.2. Total Protein and Soluble Protein Fraction 

Using CHN combustion elemental analysis the total carbon, hydrogen, and nitrogen contents in DB and RJ were compared (Table 3). Based on total nitrogen assayed by Dumas method, the total protein content was calculated using a 6.25 multiplier. An insignificant difference between DB and RJ was found (*p* > 0.05). Moreover, protein content in DB was not changed between the tested stages of development. The comparative analysis of drone brood and royal jelly soluble protein concentration was determined by the Bradford method based on the formation of a dye–protein complex (Table 3). Despite the increase in the development stage of drone brood larvae, the content of water-soluble proteins was significantly lower than that of royal jelly (*p* < 0.05). 

#### Proteomic Identification of Drone Brood and Royal Jelly Using SDS PAGE

**Figure 2:**
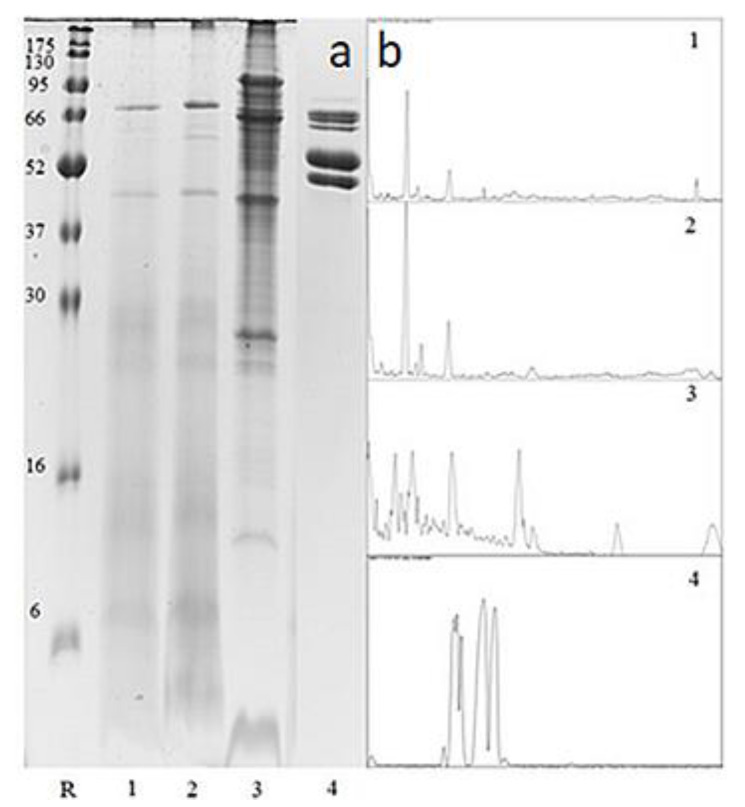
Gels obtained after protein SDS-PAGE of selected drone brood and royal jelly sample (**a**) and line plots of the respective gel lines generated with program ImageJ (1.52a) software (**b**). R-BlueEasy Prestained Protein Ladder (Nippon Genetics Co., Ltd., Tokyo, Japan)—protein molecular weight marker (kDa); (1) 7-day-old drone brood, (2) 11-day-old drone brood; (3) 14-day-old drone brood; (4) royal jelly.

A series of electrophoretic separations were carried out for selected specimens of drone brood at various stages of development and royal jelly. The analysis was carried out on samples of drone brood and royal jelly showing the best results in previous analyses (Figure 2).

The obtained protein maps (Figure 2) clearly show that the proteome of the drone brood depended on the stage of development and was completely different from the royal jelly protein profile. All three drone brood samples (regardless of development stage) have a distinct band at the level of approx. 28 and 12 kDa and 14-day-old drone brood were clearly distinguished by the occurrence of a specific band of 8 kDa. On the other hand, royal jelly contains six major protein fractions with a weight range of 50–90 kDa. 

### 3.3. Biological Activity 

#### 3.3.1. Antioxidant Activity Determination

The analysis of the antioxidant and hormonal activity of aqueous extracts of drone brood and royal jelly was carried out using the DPPH and FRAP methods (Table 4). 

The analysis of the DPPH radical scavenging capacity showed on average 27% higher activity of the drone brood compared to royal jelly, in the same concentration (10 mg/mL). A similar relationship was found by testing the activity with the FRAP method. Drone brood was more than 50% active than that of the royal jelly. Using both, DPPH and FRAP methods, the highest values were obtained for 11-day-old DB, which is in agreement with our earlier study [24].

The mean total phenolic compounds content in drone brood was 25% higher than that of royal jelly and the highest value was obtained for 7-day-old DB which slightly decreased in later stages of larvae development. In the 14-day-old brood, the content of phenolic compounds decreased significantly (*p* < 0.05).

Among the tested samples, royal jelly showed a higher content of flavonoids compared to the average content in brood by 21.89%, however, in the 11-day-old brood the highest content of flavonoids was found. The differences in the content of flavonoids in DB at different stages of development were significant (*p* < 0.05).

#### 3.3.2. Hormonal Activity

Steroid hormones (testosterone and estradiol) in drone brood and royal jelly were analyzed in this study (Table 4). 

The conducted research showed that drone brood is a valuable product containing on average 4.42 nmol/100 g of testosterone, which is 6-fold higher than royal jelly. Moreover, the testosterone level increases significantly with the development phase of the drone brood (*p* < 0.05). The 14-day-old DB contains 57% more male sex hormone than 11-day-old brood and 91.8% more than 7-day-old brood.

An opposite relationship was demonstrated by analyzing the level of estradiol in tested bee products. A comparative analysis of drone brood and royal jelly showed that DB at the earlier stages of development is a richer source of estradiol than royal jelly. On average, drone brood has over 76% higher estradiol content compared to royal jelly (*p* < 0.05). As the brood matured, the level of this hormone decreased and on day 14, the concentration was 63% lower compared to day 7 of development.

#### 3.3.3. Enzyme Activity

**Figure 3:**
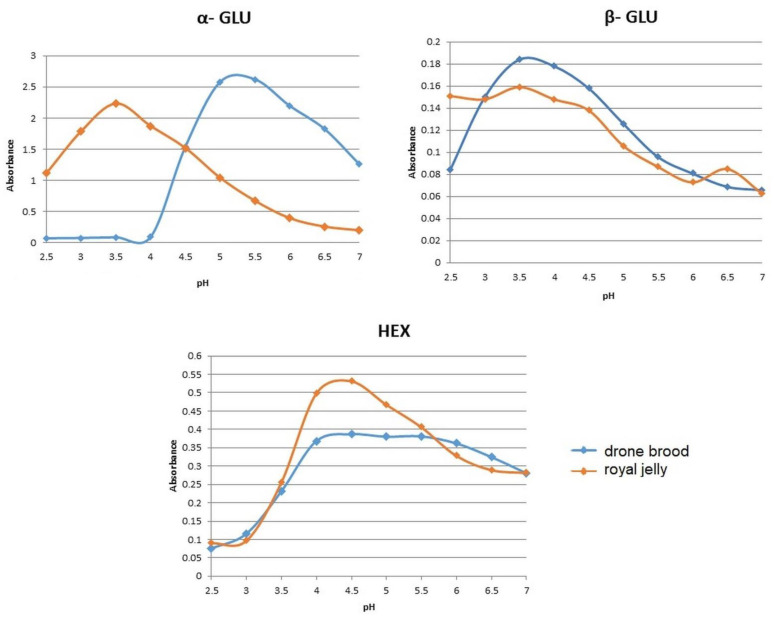
The comparison of optimum pH for selected glycosidases from drone brood and royal jelly.

Acid glycosidases were tested in drone brood for the first time. Since the enzymes required an appropriate pH to maintain catalytic activity, the optimal pH for each enzyme was tested at the beginning. Figure 3 shows the dependence of the activity of selected glycosidases on pH (in the range of pH 2.5–7.0). The calculated values of pH optimum are shown in Table 5. The highest enzyme activities were observed in the pH range of 4.0–5.0. The same pH was determined as optimal for all tested enzymes that occurred in DB and RJ, excluding β-glucosidase activity where the most acidic pH optimum was found in RJ compared to DB (Figure 3). 

Using determined optimal pH conditions, the specific activities of the tested glycosidases, as well as acid and alkaline phosphatases, were compared using the spectrophotometric method (Table 5).

Based on the tests of enzymatic activity of DB and RJ, it was found that both bee products exhibit the activity of all tested enzymes. However, royal jelly showed significantly lower enzymatic activity compared to the drone brood (*p* < 0.05). In terms of decreasing activity, the glycosidases in DB can be arranged in the following order: HEX> α-GLU> α-MAN> β-GLU> β-GAL> acid phosphatase, whereas for other studied enzymes only trace activity was found. Moreover, the peak of activity for 11-day-old brood was observed for all tested enzymes. In the case of RJ, the enzyme activity decreased in a completely different order: β-GLU> acid phosphatase> β-MAN. For HEX, the most active acid glycosidase in DB, the activity recorded in RJ was by 20-times lower. The same relationship for α-MAN was observed but when younger brood was taken for comparison only. RJ was more abundant in alkaline phosphatase, whereas DB exhibited higher activity of acid phosphatase. Moreover, for the activity of α-amylase, an enzyme that breaks down polysaccharides into simple sugars, only trace activity was demonstrated in RJ, whereas DB showed a strong activity of this enzyme. The highest amylase activity in the 7-day-old DB was checked which decreased during the development progress.

**Figure 4:**
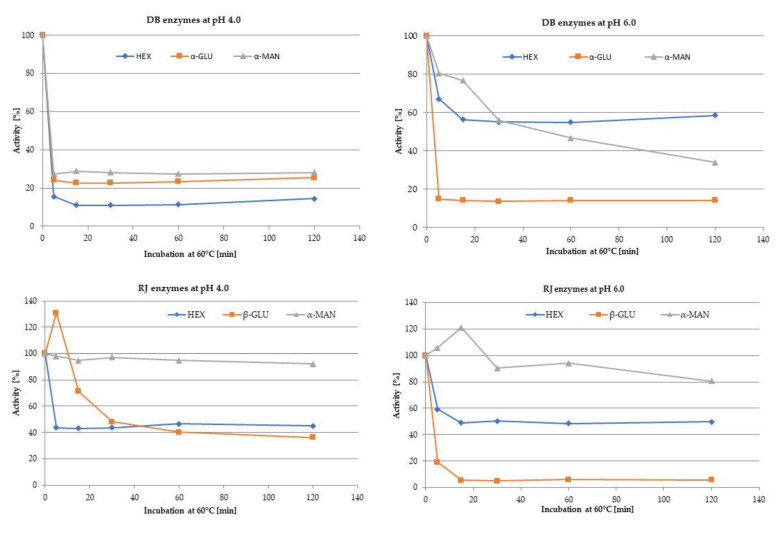
Thermostability of selected enzymes of drone brood (hexosaminidase, α-glucosidase, α-mannosidase, acid phosphatase) and royal jelly (hexosaminidase, β-glucosidase, α-mannosidase, acid phosphatase) at pH 4.0 or 6.0 during 120 min incubation at 60 °C.

Taking into account different origins of DB (tissue) and RJ (secretion) enzymes their thermal stability was tested at 60 °C, during 120 min. As the catalytic activity of an enzyme is influenced by pH, the effect of high temperature on selected enzymes activity was tested in two various pH environments (4.0 and 6.0) (Figure 4).

The analysis showed that the enzymes of the drone brood are more stable at pH 6.0 which is the natural pH of DB homogenate. In an acidic environment, they were losing their activity quickly. For RJ enzymes, the stabilizing effect of less acidic pH on thermal inhibition of tested enzyme activity was not observed, on the contrary, the activity of β-glucosidase was completely destroyed at this pH. 

### 3.4. High Performance Thin Layer Chromatography Analysis

#### 3.4.1. Polyphenolic Profile

**Figure 5:**
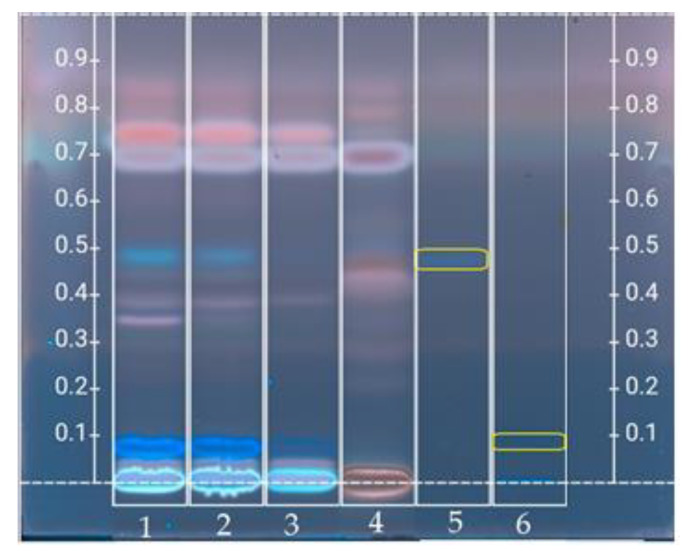
HPTLC chromatogram of phenolic compounds at 365 nm (after p-anisaldehyde derivatization) in crude drone brood and royal jelly, where (1) 7-day-old; (2) 11-day-old; (3) 14-day-old; (4) royal jelly; (5) ferulic acid; (6) ellagic acid. (Drone brood and royal jelly extracts showing the best results in previous analyses were only shown).

The chromatogram of the separation of phenolic acids and flavonoids in the crude drone brood homogenates and royal jelly is shown in Figure 5. The bands create unique fingerprints of drone brood and royal jelly and allow for visual differentiation, forming a system similar to common barcodes used for marking consumer goods. 

The HPTLC phenolic profile chromatogram (Figure 5) is characterized by differentiation in terms of the location of the bands and their color intensity, which proves qualitative and quantitative differences in the analyzed samples of drone brood and royal jelly. The identification of the phenolic compounds was based on the Rf values (distance) and color of the standards used. The obtained profiles contain mainly blue bands (characteristic for caffeic, p-coumaric, and ferulic acid) [25]. Blue bands dominate in the DB homogenate chromatogram, which proves the presence of ferulic acid (Rf = 0.48) and ellagic acid (Rf = 0.08) in the tested material. Additionally, some white stained bands are visible in the DB samples (at Rf = 0.39, 0.34 for a 7-day DB). In contrast, the RJ sample shows numerous bands of slightly pink color. The group of bands with high Rf values (above 0.65) stained pink or purple corresponds most likely to steroid compounds.

HPTLC chromatograms show similarities and differences in the composition of the identified compounds between the drone brood and royal jelly, as well as within the different development stages of the drone brood. Based on the intensity of the band, it should be concluded that in 14-day-old male brood much smaller amounts of ferulic acid were found, while ellagic acid was present in trace amounts. Moreover, no bands were found in the royal jelly to indicate the presence of ferulic and ellagic acids.

#### 3.4.2. Sugars

During the HPTLC separation of reducing sugars, after derivatization and heating the plate to 110 °C for 10 min, gray-green bands are formed on an almost colorless background, and some of them fluoresce at 365 nm, which is crucial for the construction of characteristic fingerprints [26].

**Figure 6:**
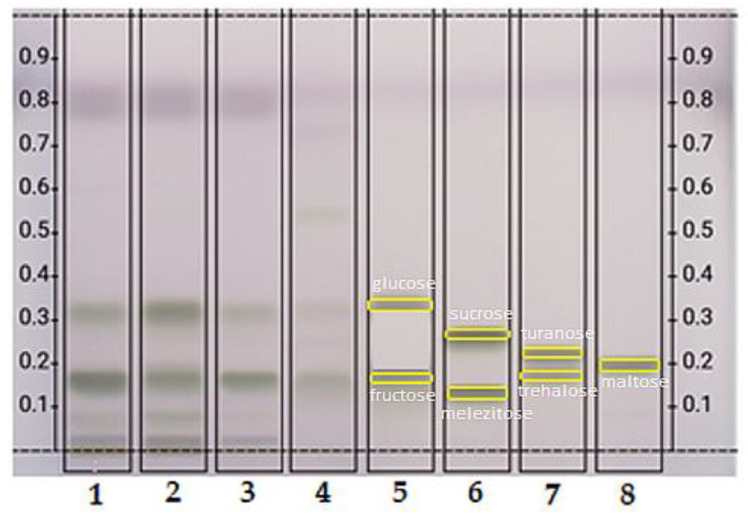
HPTLC chromatogram of sugars compounds at RT white in crude drone brood and royal jelly, where (1) 7-day-old; (2) 11-day-old; (3) 14-day-old; (4) royal jelly; (5) glucose, fructose; (6) sucrose, melezitose; (7) trehalose, turanose; (8) maltose. (Drone brood and royal jelly extracts showing the best results in previous analyses were only shown).

Figure 6 shows the sugar profile of drone brood and royal jelly. The reference standards used were glucose (Rf = 0.34), fructose (Rf = 0.24), sucrose (Rf = 0.30), melezitose (Rf = 0.17), trehalose (Rf = 0.21), turanose (Rf = 0.26), maltose (Rf = 0.22). The sugar bands in the drone brood were more intense and more pronounced, which proves the higher content of the discussed compounds. Based on the intensity of the bands, it was found that drone brood is a richer source of glucose and fructose compared to royal jelly. Moreover, the intensity of the bands indicated that the content of the analyzed reducing sugars in the brood varies regardless of the stage of development. 

Royal jelly contains fewer reducing sugars analyzed, as demonstrated by the weak intensity of the glucose and fructose bands. Moreover, the presence of trehalose in the brood and royal jelly cannot be eliminated, because its Rf is very similar to the fructose Rf, so that the bands may overlap, which makes it difficult to accurately identify the sugars in the bee products tested. However, it should be noted that the royal jelly chromatogram shows two additional bands of weak intensity. The first at the height of turanose (Rf = 0.26) and the second undefined at Rf = 0.53. Similarly, additional bands for DB samples which did not occur in RJ profile were noted at Rf = 0.08 and 0.01. These compounds have not been identified, which indicates the need for further, extensive research.

#### 3.4.3. Amino Acids

**Figure 7:**
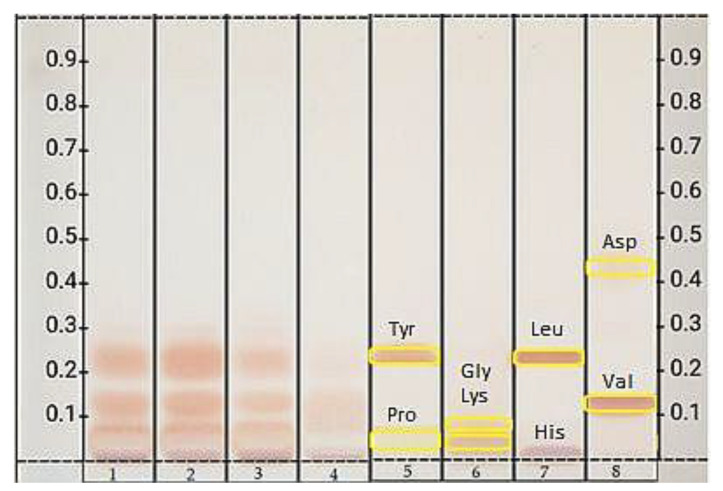
HPTLC chromatogram of amino acids compounds at RT white in crude drone brood and royal jelly, where (1) 7-day-old; (2) 11-day-old; (3) 14-day-old; (4) royal jelly; (5) proline, tyrosine; (6) glycine, lysine; (7) histidine, leucine; (8) aspartic acid, valine. (Drone brood and royal jelly extracts showing the best results in previous analyses were only shown).

The chromatographic results of amino acids fingerprints determination in drone brood in different stages of development and royal jelly are presented in Figure 7.

The HPTLC chromatograms indicate similarities and differences in the composition of the identified compounds between drone brood and royal jelly. The bands for drone brood are dominated by yellowish and orange color, with major bands at Rf in the range of 0.01–0.30. The chromatogram bands for royal jelly are much less intense in color compared to drone brood, which indicates a lower content of amino acids. However, the royal jelly presents notable bands at Rf in the same range as in drone brood. Proline (Rf = 0.04) which forms light yellow to white band, was present in the RJ. The band is also visible in the drone brood, however, due to the overlapping of other amino acids with a similar Rf (glycine Rf = 0.07; lysine Rf = 0.08) the proline band was poorly visible. Moreover, a band with an Rf = 0.29, which is assigned to tyrosine and/or leucine, as well as valine (Rf = 0.15), was also identified in the brood. A poorly visible valine band was also found in royal jelly. The histidine band (left on the starting line) was present in all samples in a higher amount in DB than RJ.

## 4. Discussion

For the first time, drone brood and royal jelly that originated from the same apiaries were compared in terms of their chemical composition. Moreover, the changes in chemical composition of drone brood during larvae development were tested. As DB and RJ are assumed to have a similar chemical composition, recent cases of adulteration of RJ with the addition of brood were detected. This is because DB is an easy to obtain bee product compared to RJ only [27]. Obtaining drone brood on an industrial scale is easier and more beneficial than royal jelly. Drone larvae can be extracted easily from the combs before capping even with the use of a stream of water. Moreover, up to 1.3 kg of drone brood can be obtained annually on average from one bee family without consequences for its condition. In opposite, royal jelly can be obtained in larger quantities only in specialized apiaries, where it is produced using royal jelly-producing chamber [25].

The comparison between RJ and DB was started from basic physicochemical parameters analysis. All tested parameters were found to be stable in drone brood regardless of the stage of larvae development. DB contained more water than royal jelly. Our finding of the moisture content for drone brood was similar to earlier reported data [28,29,30]. Royal jelly was more acidic but exhibited lower total acidity compared to DB. Obtained data agreed with the values reported by Krell [31], Ramadan and Al-Ghamdi [8], and Nabas et al. [32]. The observed differences in pH and acidity values could be a useful tool in the detection of RJ adulteration with drone brood. 

Both tested bee products were found as valuable sources of macro- and microelements, however, in most cases, the royal jelly was more abundant in minerals. Any significant differences resulting from botanical and geographic origins of samples were observed. This fact emphasizes the homeostatic regulation of the concentrations of trace and mineral elements produced in the endocrine glands of bees [3]. In turn, in the course of larvae development great fluctuations in the content of bioelements were observed. Higher phosphorus but a much lower calcium content was found in DB than in RJ. Among microelements, iron and manganese prevailed in DB, whereas higher zinc level was found in RJ. The results obtained in this work are in line with other authors’ studies. Stocker et al. [33] checked the similar mineral composition of royal jelly. It showed that the main element was potassium (246.6–312 mg/100 g), phosphorus (194–235 mg/100 g), calcium (113–145 mg/100 g) and magnesium (26.4–31.2 mg/100 g). Similar results were reported for royal jelly by Bengu et al. [34]. Moreover, the results obtained for the drone brood are in agreement with available literature. Prokhoda et al. [35] showed a similar content of magnesium, manganese, and copper in the analyzed DB samples.

Similar protein content was found in drone brood and royal jelly. Moreover, the protein level was not dependent on the developmental stage of DB. On the opposite, when only soluble protein fraction was analyzed, the higher value for RJ and the increase of protein content in the course of larvae development were found. The obtained DB total proteins content was consistent with the literature data [35,36]. The value found for RJ samples in this study was within the range reported by several authors [8,32,36,37]. The water-soluble proteins accounted for only 25–35% of the total protein content in the drone brood and 50% in the royal jelly. This is because the Dumas method measures the protein content indirectly based on its nitrogen content which includes other biomolecules containing nitrogen also, e.g., amino acids, nucleic acids, and other nitrogenous compounds.

The protein composition of the DB was more diversified than RJ. The proteome of the drone brood was related to the stage of development and was completely different from the royal jelly protein profile, especially in the range of lower-mass proteins. For royal jelly six major protein fractions with a mass range of 50–90 kDa were detected, which corresponds to MRJP proteins. The most intense band has approx. 57 kDa, which corresponds to MRJP-1 [38]. The observed differences seem to be obvious considering the origin of both samples: DB as a tissue sample contains other proteins that additionally differentiate as the larva develops, while RJ is a bee secretion with a stable composition.

Among proteins detected in tested bee products, the most important are enzymes, including lysosomal glycosidases which belong to glycoproteins [4,5]. Most of these enzymes, characterized by acidic pH optimum, are common in various tissues of animals and can be secreted outside the cell into body fluids [4]. Thus, their occurrence has been expected in both drone larvae and royal jelly extracts. However, different enzymatic profiles for both materials were found. Drone brood showed high hexosaminidase activity (22 times higher than RJ) which is typical for tissue homogenates [39,40]. The most active were α-glucosidase, α-mannosidase and β-galactosidase specific for tissue extracts. Whereas, for royal jelly β-glucosidase and β-mannosidase were found as the most active. Testing the dependence of the activity of tested glycosidases on pH (in the range 2.5–7.0), it was shown that they exhibited the pH optimum in the range of 4.0–5.0 the same for DB and RJ, excluding β-glucosidase from RJ. The activity of these enzymes was studied in DB for the first time and the results are in line with those obtained for RJ [4]. When the temperature increased above 40 °C, gradual denaturation and loss of catalytic abilities were noted. However, the pH environment affected the thermal resistance of DB enzymes only. 

Droba et al. [4,5] investigated the activity of acid glycosidases in royal jelly and queen bee larvae. They showed many times greater enzyme activity of queen bee larvae than royal jelly. Moreover, the activity of enzymes in individual samples of royal jelly was varied. Amylolytic and glycolytic enzymes can enrich the environment with easily digestible nutrients and other valuable ingredients. On the other hand, lysosomal glycosidases may favor the processes taking place in the royal jelly modifying the composition of the glycan component of glycoconjugates [5]. Diastase is one of the major enzymes found in honey. Diastase activity is well used as a criterion to assess the quality of the product [41]. However, to date, no studies have been found showing α-amylase activity in brood or royal jelly. The analysis showed high α-amylase activity in drone brood, while in royal jelly this enzyme was present in trace amounts.

Both tested bee products exhibited antioxidant properties, however, DB was more active than RJ, regardless of the assay. The antiradical properties of drone brood were changing during larvae development. Thus, the highest values obtained by DPPH and FRAP methods for 11-day-old DB in this study are in line with our earlier report [22]. Drone brood antioxidants are represented by polyphenolic compounds, vitamins C and E, enzymes, unsaturated fatty acids in free and bound forms, including unique decenoic acids, substances having sulfhydryl groups, flavonoid, and other phenolic compounds [42]. Silici [13] examined the bioactivity of apilarnil (lyophilized drone brood) and showed the total phenolic content at the level 834.05 mg GAE/100 g which is comparable with the results of this work when calculated them to dry mass. 

Direct visual comparison of reducing sugar bands obtained by HPTLC analysis for brood and royal jelly with the standards entered, provided new reliable semi-quantitative information. It should be emphasized that the TLC method is most suitable for detecting many carbohydrates present in food products, mainly in fruit and vegetable juices [43]. The obtained data are in line with those presented by Bogdanov [12], who proved that drone brood contains two times more glucose than royal jelly. Similarly, Margaoan et al. [44] checked the carbohydrate content in royal jelly and drone brood using the HPLC-IR method and showed a slightly higher content of glucose (1%) than fructose in royal jelly. The same correlation was observed for the drone brood, but the glucose content was several hundred times higher than that of fructose [44]. Moreover, in royal jelly, he showed sucrose, which was not found in the brood. Drone brood was a richer source of maltose and trehalose compared to royal jelly [44].

This study presented also a new idea to test the amino acid profile in bee products using HPTLC analysis. The chromatogram bands for royal jelly amino acids were much less intense compared to drone brood, which indicates a lower content of amino acids. However, the royal jelly presents notable bands at Rf in the same range as in drone brood, including proline, glycine, lysine, tyrosine, and leucine, as well as valine, which was the most intense band in the 11-day-old brood. Obtained data are in agreement with other author findings where the greatest amounts of glutamic acid, leucine and aspartic acid, proline, lysine, valine, and alanine which make up about 60% of all amino acids of drone brood and royal jelly [27,44]. Amino acids that could be markers of the quality of royal jelly and drone brood are mainly glutamine—found only in royal jelly and threonine present in much greater amounts in drone brood. A similar relationship was observed for leucine, histidine, and tyrosine [44]. In turn, drone brood has the highest content of proline from all the analyzed bee products and for this reason, this bee product is very important as a food supplement or an ingredient in other types of natural supplements [45].

Based on the analysis of the color intensity and the number of bands on HPTLC chromatograms, it should be concluded that the richest source of phenolic compounds is the 7-day-old drone brood, and the weakest is the 14-day-old one. However, the phenolic profiles obtained for drone brood and royal jelly were completely different, ferulic and ellagic acids were not found in royal jelly. Such observation was done for the first time. According to earlier reports, some flavonoids were expected [24,34,46] and tested (naringenin, apigenin, chrysin, sakuranetin) there are certain flavonoids: flavanones (hesperetin, naringenin), flavones (apigenin derivatives), and isoflavonoids (genistein, formononetin, coumestrol), as well as ferulic acid present in royal jelly. However, there are no polyphenol profiles available for the drone brood. Differences in phenolic compound profiles in the future could be used to determine adulteration of royal jelly with drone brood in order to improve quality. Due to the limited availability of reference substances and the unknown topic, further analyses are needed to fully identify the unknown compounds.

In some population cultures, honeybee larvae are used to treat male infertility [45,47]. Previously, it was mistakenly assumed that the use of larvae for infertility treatment was due to the high protein content in the larvae, but subsequent chemical analysis revealed the presence of sex hormones in drone larvae [48]. The obtained results of the hormonal activity of testosterone and estradiol in drone brood (in the early stage of development) and royal jelly are comparable with the Bogdanov [12], who found that the crude brood homogenate contains 0.31 nmol/100 g of testosterone, 51.3 nmol/100 g of progesterone, 410.65 nmol/100 g of prolactin, and 677.6 nmol/100 g of estradiol. Moreover, crude royal jelly contains 0.20 nmol/100 g of testosterone and 52.0 nmol/100 g of estradiol [12]. It should also be stated that the early stage of drone brood is more active in testosterone and estradiol than RJ. As the stage of development changes, the activity of testosterone increases and estradiol decreases. Since royal jelly showed much lower hormonal activity—both testosterone and estradiol—testing the hormonal activity may be a checkpoint for the adulteration of royal jelly with drone brood.

## 5. Conclusions

Drone brood, considered the male equivalent of royal jelly, is a new, little known and rarely tested bee product. The chemical composition related to the drone larvae developmental stage has not been studied in detail yet. It was found that the content of bioactive compounds changed during larvae development and the highest level of the majority of tested parameters for 11-day-old brood was observed. However, the change in the content of bioactive compounds caused by developmental changes, drone brood, compared to royal jelly, shows better physicochemical properties and greater biological activity, mainly hormonal, enzymatic, and antioxidant.

Due to the high physicochemical similarity, royal jelly may be admixed with drone brood, which is not officially marketed as a food product or food additive. Thus, searching for effective markers to differentiate adulterated royal jelly seems to be an urgent problem. As the most promising indicators of royal jelly purity, α-amylase activity together with other glycosidases (hexosaminidase, α-glucosidase, and α-mannosidase) were selected.

Drone brood as an easy to obtain beekeeping product, sometimes considered as waste, could be applied as a direct functional food thanks to the greater abundance of protein, amino acid, steroids and polyphenols compared to royal jelly. 

## Figures and Tables

**Table 1:** Details of separations performed on HPTLC Silica gel 60 F_254_.

Identified Group	Mobile Phase (v:v:v)	Derivatization Reagent	Used Standards
Polyphenolics	Chloroform:ethyl acetate:formic acid (5:4:1)	p-anisaldehyde-sulfuric acid reagent	Ferulic acid, ellagic acid
Sugars	1-propanol:1-butanol:boric acid 5 mg/mL H_2_O (3:5:1)	p-anisaldehyde-sulfuric acid reagent	Glucose, fructose, trehalose, melezitose, sucrose, turanose, maltose
Amino acids	1-butanol:acetic acid:water (3:1:1)	Ninhydrin in ethanol (2 mg/50 mL)	Proline, tyrosine, glycine, lysine, histidine, leucine, aspartic acid, valine

**Table 2:** Physicochemical properties and mineral composition of drone brood (7-, 11-, 14-day-old) and royal jelly. The range and mean value for three samples obtained from various apiaries are shown.

	Physicochemical Composition Min–Max (Mean)				
	Drone Brood				Royal Jelly (*n* = 3)
	7-day-old (*n* = 3)	11-day-old (*n* = 3)	14-day-old (*n* = 3)	Mean (7–14-day-old)	
**Water content [%]**	69.6–70.3 (69.9) ^a^	69.4–72.8 (71.6) ^a^	68.7–73.1 (71.9) ^a^	71.1 ^A^	65.4–69.0 (68.4) ^aA^
**pH**	6.52–6.58 (6.53) ^b^	6.62–6.70 (6.66) ^b^	6.58–6.78 (6.63) ^b^	6.60 ^A^	3.97–3.98 (3.97) ^aB^
**Acidity [mval/g WW]**	10.0–18.0 (13.3) ^b^	10.0–18.5 (14.3) ^b^	9.90–19.0 (14.4) ^b^	14.0 ^B^	6.30–6.50 (6.36) ^aA^
**Conductivity [mS/cm]**	0.03–0.04 (0.03) ^a^	0.03–0.04 (0.03) ^a^	0.03–0.04 (0.03) ^a^	0.03 ^A^	0.03–0.04 (0.03) ^aA^
**Refractive** **index [n_D_]**	1.337–1.339 (1.338) ^a^	1.337–1.339 (1.338) ^a^	1.337–1.339 (1.338) ^a^	1.338 ^A^	1.340–1.342 (1.341) ^aA^
	**Macroelements min–max (mean) [mg/100 g WW]**				
**Na**	6.45–10.27 (8.73) ^b^	8.63–26.09 (6.99) ^a^	7.32–9.93 (8.40) ^b^	8.04 ^A^	10.3–13.8 (12.0) ^cB^
**K**	276.06–337.06 (287.32) ^a^	240.96–313.99 (286.36) ^a^	259.30–314.32 (292.58) ^a^	288.75 ^A^	321.1–357.4 (391.0) ^bB^
**Ca**	15.16–21.02 (17.77) ^b^	8.91–57.05 (27.98) ^d^	8.13–17.47 (12.52) ^a^	19.42 ^A^	22.8–24.0 (23.5) ^cB^
**Mg**	29.21–34.02 (32.04) ^a^	15.25–33.24 (26.18) ^a^	21.02–122.73 (56.57) ^c^	38.26 ^A^	44.0–50.4 (47.2) ^bB^
**P**	296.69–324.02 (302.40) ^a^	212.22–348.70 (287.92) ^a^	267.26–351.35 (316.21) ^b^	302.17 ^A^	338.4–412.1 (375.0) ^cB^
**S**	392.37–99.27 (96.30) ^b^	53.33–100.99 (75.85) ^a^	99.27–126.87 (110.39) ^c^	94.18 ^A^	153.2–169.3 (161.2) ^dB^
	**Microelements min–max (mean) [mg/100 g WW]**				
**Fe**	1.17–1.23 (1.20) ^b^	0.57–1.17 (0.93) ^a^	1.29–1.62 (1.41) ^c^	1.18 ^A^	n.d.
**Mn**	0.21–0.45 (0.33) ^c^	0.27–0.33 (0.21) ^b^	0.06–0.39 (0.18) ^a^	0.24 ^B^	0.01–0.08 (0.05) ^aA^
**Zn**	41.44–1.71 (1.56) ^b^	0.89–1.80 (1.29) ^a^	1.14–2.04 (1.49) ^ab^	1.44 ^A^	2.07–2.58 (2.32) ^cB^
**Cr**	0.01–0.03 (0.02) ^a^	0.00–0.03 (0.01) ^a^	0.02–0.07 (0.04) ^a^	0.02 ^A^	0.03–0.15 (0.09) ^aA^
**Cu**	0.29–0.44 (0.36) ^b^	0.15–0.39 (0.27) ^a^	0.32–0.38 (0.35) ^b^	0.32 ^A^	0.31–0.39 (0.33) ^abA^
	**Other min–max (mean) [mg/100 g WW]**				
**Sr**	0.00–0.006 (0.004) ^a^	0.00–0.18 (0.06) ^a^	0.00–0.006 (0.003) ^a^	0.022 ^A^	n.d.
**As**	0.00–0.002 (0.03) ^a^	0.00–0.006 (0.003) ^a^	0.003–0.003 (0.003) ^a^	0.003 ^A^	0.01–0.09 (0.04) ^aB^
	**Contaminants min–max (mean) [mg/100 g WW]**				
**Al**	0.39–0.64 (0.48) ^a^	0.48–1.19 (0.76) ^b^	0.54–1.15 (0.93) ^c^	0.62 ^A^	0.48–3.35 (0.64) ^bA^
**Cd**	0.003–0.003 (0.003) ^a^	0.00–0.007 (0.003) ^a^	0.00–0.003 (0.0003) ^a^	0.0021 ^A^	n.d.
**Pb**	0.00–0.005 (0.003) ^a^	00.00–0.004 (0.004) ^a^	0.003–0.006 (0.003) ^a^	0.0033 ^A^	0.04–0.07 (0.02) ^aA^

^a,b,c,d^—results marked with a different superscript in the rows are significantly different (Tukey’s test, *p* < 0.05). ^A,B^—results marked with a different superscript between royal jelly and mean value of drone brood are significantly different (Tukey’s test, *p* < 0.05).

**Table 3:** Results of CHN analysis, calculated total protein content and soluble protein content measured by Bradford method for drone brood and royal jelly.

	Drone Brood				Royal Jelly (*n* = 3)
	7–day-old (*n* = 3)	11-day-old (*n* = 3)	14-day-old (*n* = 3)	Mean (7–14-day-old)	
**CHN** **elemental analysis**					
**C [%]**	16.49–16.70 (16.58) ^b^	16.49–16.60 (16.54) ^b^	15.92–16.99 (16.57) ^b^	16.56 ^B^	13.23–14.06 (13.64) ^aA^
**H [%]**	2.09–2.12 (2.11) ^a^	2.06–2.12 (2.10) ^a^	2.12–2.17 (2.14) ^a^	2.11 ^A^	2.89–2.41 (2.15) ^aA^
**N [%]**	2.08–2.25 (2.16) ^a^	2.12–2.35 (2.25) ^a^	2.01–2.34 (2.21) ^a^	2.20 ^A^	2.02–2.89 (2.45) ^aA^
**Total protein [%]**	12.52–13.53 (12.99) ^a^	12.75–14.17 (13.54) ^a^	12.09–14.09 (13.31) ^a^	13.28 ^A^	10.43–18–14 (14.12) ^aA^
**Soluble protein [%]**	3.30–4.20 (3.41) ^a^	4.19–4.57 (4.32) ^b^	4.48–4.72 (4.63) ^b^	4.12 ^A^	6.88–7.96 (7.28) ^cB^

^a,b,c^—results marked with a different superscript in the rows are significantly different (Tukey’s test, *p* < 0.05). ^A,B^—results marked with a different superscript between royal jelly and mean value of drone brood are significantly different (Tukey’s test, *p* < 0.05).

**Table 4:** Antioxidant activity (DPPH, FRAP), total phenolic (TPC), and total flavonoids as well as hormonal content of analyzed drone brood and royal jelly.

Antioxidant Activity:	Drone Brood				Royal Jelly (*n* = 3)
	7-day-old (*n* = 3)	11-day-old (*n* = 3)	14-day-old (*n* = 3)	Mean (7–14-day-old)	
DPPH [%]	9.2–16.36 (12.95) ^b^	6.91–24.76 (16.82) ^c^	10.43–16.98 (13.60) ^bc^	14.45 ^B^	7.94–11.03 (10.52) ^aA^
FRAP [µmol TE/100 g]	0.80–1.16 (0.97) ^b^	0.79–1.27 (1.04) ^b^	0.86–1.63 (1.03) ^b^	1.01 ^B^	0.18–0.20 (0.19) ^aA^
TPC [mg GAE/100 g]	234.62–268.84 (267.57) ^c^	180.05–320.43 (259.79) ^c^	200.89–285.21 (234.12) ^b^	253.82 ^B^	181.54–191.96 (189.72) ^aA^
TFC [mg/100 g]	6.71–9.92 (8.94) ^a^	10.30–13.0 (11.42) ^b^	4.12–9.80 (7.46) ^a^	9.27 ^A^	10.7–11.9 (11.3) ^bB^
**Hormonal activity**					
Testosterone [nmol/100 g]	0.47–1.10 (0.79) ^a^	3.64–3.96 (3.80) ^b^	8.24–9.10 (8.67) ^c^	4.42 ^B^	0.64–0.72 (0.68) ^aA^
Estradiol [nmol/100 g]	653.70–680.26 (664.75) ^d^	332.37–370.45 (355.67) ^c^	217.90–274.16 248.78 ^b^	426.06 ^B^	94.12–116.03 (106.46) ^aA^

^a,b,c,d^—results marked with a different superscript in the rows are significantly different (Tukey’s test, *p* < 0.05). ^A,B^—results marked with a different superscript between royal jelly and mean value for drone brood are significantly different (Tukey’s test, *p* < 0.05).

**Table 5:** Specific enzyme activity in drone brood and royal jelly measured in optimal pH conditions.

Enzyme	pH Optimum	Drone Brood [U/100 g WW]				Royal Jelly [U/100 g WW] (*n* = 3)
		7-day-old (*n* = 3)	11-day-old (*n* = 3)	14-day-old (*n* = 3)	Mean (7–14-day-old)	
α-**GLU**	5.0 DB; 3.5 RJ	47.0–47.8 (47.4) ^c^	82.2–84.1 (82.9) ^d^	19.5–22.0 (20.7) ^b^	50.3 ^B^	0.6–0.9 (0.7) ^aA^
β-**GLU**	4.0 DB; RJ	2.2–3.9 (3.0) ^ab^	3.9–5.6 (4.7) ^b^	0.00–0.8 (1.0) ^a^	2.90 ^A^	6.9–7.7 (7.4) ^cB^
α-**GAL**	4.0 DB; RJ	0.4–1.4 (0.9) ^ab^	0.8–2.3 (1.2) ^c^	0.3–1.7 (1.1) ^bc^	0.99 ^B^	0.5–0.7 (0.6) ^aA^
β-**GAL**	4.0 DB; RJ	1.6–2.4 (2.0) ^b^	2.7–4.2 (3.3) ^d^	1.2–4.1 (2.9) ^cd^	2.73 ^B^	0.6–0.7 (0.6) ^aA^
α-**MAN**	5.0 DB; RJ	21.0–22.8 (21.9) ^c^	33.0–34.4 (33.9) ^d^	2.0–4.6 (3.4) ^b^	19.71 ^B^	1.2–1.5 (1.3) ^aA^
β-**MAN**	5.0 DB; RJ	0.6–1.0 (0.8) ^a^	1.0–2.7 (1.8) ^b^	0.1–1.3 (0.7) ^a^	0.99 ^A^	0.7–0.9 (0.8) ^aA^
**HEX**	5.0 DB; RJ	59.0–68.3 (64.1) ^b^	156.8–157.7 (157.1) ^d^	80.8–88.0 (81.8) ^c^	101.0 ^B^	0.9–8.1 (4.5) ^aA^
**Alkaline** **phosphatase**	10.5 DB; RJ	0.1–1.2 (0.5) ^a^	0.1–1.4 (0.7) ^a^	0.1–1.0 (0.5) ^a^	0.56 ^A^	0.8–1.5 (1.1) ^bB^
**Acid** **phosphatase**	5.0 DB; RJ	1.6–3.4 (2.4) ^b^	3.7–5.5 (4.5) ^c^	1.0–3.7 (2.1) ^ab^	3.0 ^B^	1.5–1.6 (1.6) ^aA^
**α-amylase (U/g)**	5.0 DB; RJ	14.0–17.60 (16.4) ^d^	13.1–14.5 (13.8) ^c^	5.2–11.9 (8.15) ^b^	12.78 ^B^	0.46–0.64 (0.56) ^aA^

^a,b,c,d^—results marked with a different superscript in the rows are significantly different (Tukey’s test, *p* < 0.05). ^A,B^—results marked with a different superscript between royal jelly and mean value for drone brood are significantly different (Tukey’s test, *p* < 0.05).

## Data Availability

Data is contained within the article.
